# Liquid-Based Iterative Recombineering Method Tolerant to Counter-Selection Escapes

**DOI:** 10.1371/journal.pone.0119818

**Published:** 2015-03-16

**Authors:** Masahiro Tominaga, Shigeko Kawai-Noma, Ikuro Kawagishi, Yoshiyuki Sowa, Kyoichi Saito, Daisuke Umeno

**Affiliations:** 1 Department of Applied Chemistry and Biotechnology, Faculty of Engineering, Chiba University, 1-33 Yayoi-Cyo, Inage-ku, Chiba 263-8522, Japan; 2 Department of Frontier Bioscience, Hosei University, 3-7-2, Koganei, Tokyo 184-8584, Japan; 3 Research Center for Micro-Nano Technology, Hosei University, 3-11-15 Midori-cho, Tokyo 184-8584, Japan; 4 Precursory Research for Embryonic Science and Technology (PRESTO), Japan Science and Technology Agency (JST), 4-1-8 Honcho, Kawaguchi, Saitama 332-0012, Japan; Imperial College London, UNITED KINGDOM

## Abstract

Selection-based recombineering is a flexible and proven technology to precisely modify bacterial genomes at single base resolution. It consists of two steps of homologous recombination followed by selection/counter-selection. However, the shortage of efficient counter-selectable markers limits the throughput of this method. Additionally, the emergence of ‘selection escapees’ can affect recombinant pools generated through this method, and they must be manually removed at each step of selection-based recombineering. Here, we report a series of efforts to improve the throughput and robustness of selection-based recombineering and to achieve seamless and automatable genome engineering. Using the nucleoside kinase activity of herpes simplex virus thymidine kinase (hsvTK) on the non-natural nucleoside dP, a highly efficient, rapid, and liquid-based counter-selection system was established. By duplicating *hsvtk* gene, combined with careful control of the population size for the subsequent round, we effectively eliminated selection escapes, enabling seamless and multiple insertions/replacement of gene-size fragments in the chromosome. Four rounds of recombineering could thus be completed in 10 days, requiring only liquid handling and without any need for colony isolation or genotype confirmation. The simplicity and robustness of our method make it broadly accessible for multi-locus chromosomal modifications.

## Introduction

Recombination-mediated genetic engineering, known as recombineering, is an efficient and flexible method for modifying host genomes, permitting researchers to delete, replace, and insert DNA at any targeted chromosomal site at single base resolution. In particular, the system using phage-based machinery (Lambda RED recombination system [1]) has been widely used because of its simplicity and flexibility. Because of the low frequency of homologous recombination of gene-size fragments (at most 10^−4^ recombinants per viable cell, even with the aid of the Lambda RED system [2]), two steps of homologous recombination followed by selection/counter-selection [3] are required. One of the issues of this selection-based recombineering is the shortage of convenient counter-selectable markers. As of today, all accessible counter-selectable markers require either solid media [4,5] or special mutant alleles [6–11].

Another problem with counter-selection is that the counter-selectable markers develop mutations, either by PCR amplification or by spontaneous mutations inside the cells, which inactivate the counter-selectable marker genes or generate mutant cells that are immune to the toxic effect of the counter-selectable marker [11]. Once such ‘counter-selection escape’ mutants emerge, they can quickly dominate the cell population and thereby preclude the enrichment of recombinants in subsequent rounds. The yield of recombinants (10^−5^ to 10^−4^/cell, depending on the context and length of the homology arms, it could be as low as 10^−7^/cell) is not much higher than or sometimes even lower than that of the emergent false-positives (10^−6^ to 10^−5^/cell). Consequently, experimentalists must manually pick multiple colonies and analyze their genotypes to isolate the correct clones in each round of selection-based recombineering.

With the maturation of synthetic and systems biology, there is a growing demand for genome modification technologies with more scalable and combinatorial nature. Emerging technologies such as Multiplex Automated Genome Engineering (MAGE) [12,13], Transcription Activator-Like Effector Nucleases (TALEN) [14], and Clustered Regularly Interspaced Short Palindromic Repeat (CRISPR) [15] are enabling researchers to modify chromosomes at an unprecedented scale. Notably, MAGE takes advantage of the ultra-high efficiency (0.1–0.3 events/cell [12]) in oligonucleotide-directed short-patch recombination to achieve simultaneous editing of multiple targets in a fully automated manner; given its selection-free format, experimentalists do not need to search for reliable selectable markers or continually combat selection escape. The applications of this technology range from the fast-track creation of a semi-rational chromosomal library (∼10^10^) in a search for isoprenoid hyper-producers [12] and the simultaneous incorporation of T7 promoters into 12 relevant genes [13], to the addition of histidine tags to 38 targeted genes [16] and the simultaneous replacement of hundreds of codons [17–19] spread over the genome. In theory, a conventional two-step selection-based recombineering method could be repeated for multiple cycles as well, if the aforementioned technical problems are solved. Given that two-step selection-based recombineering is a widely used and flexible technology, and given the lack for automatable multiplex genome engineering technology for gene-size fragments [20], we aimed to greatly improve the convenience, rapidity, and robustness of this method to enable the parallel and continuous operation of genome engineering.

**Fig 1:**
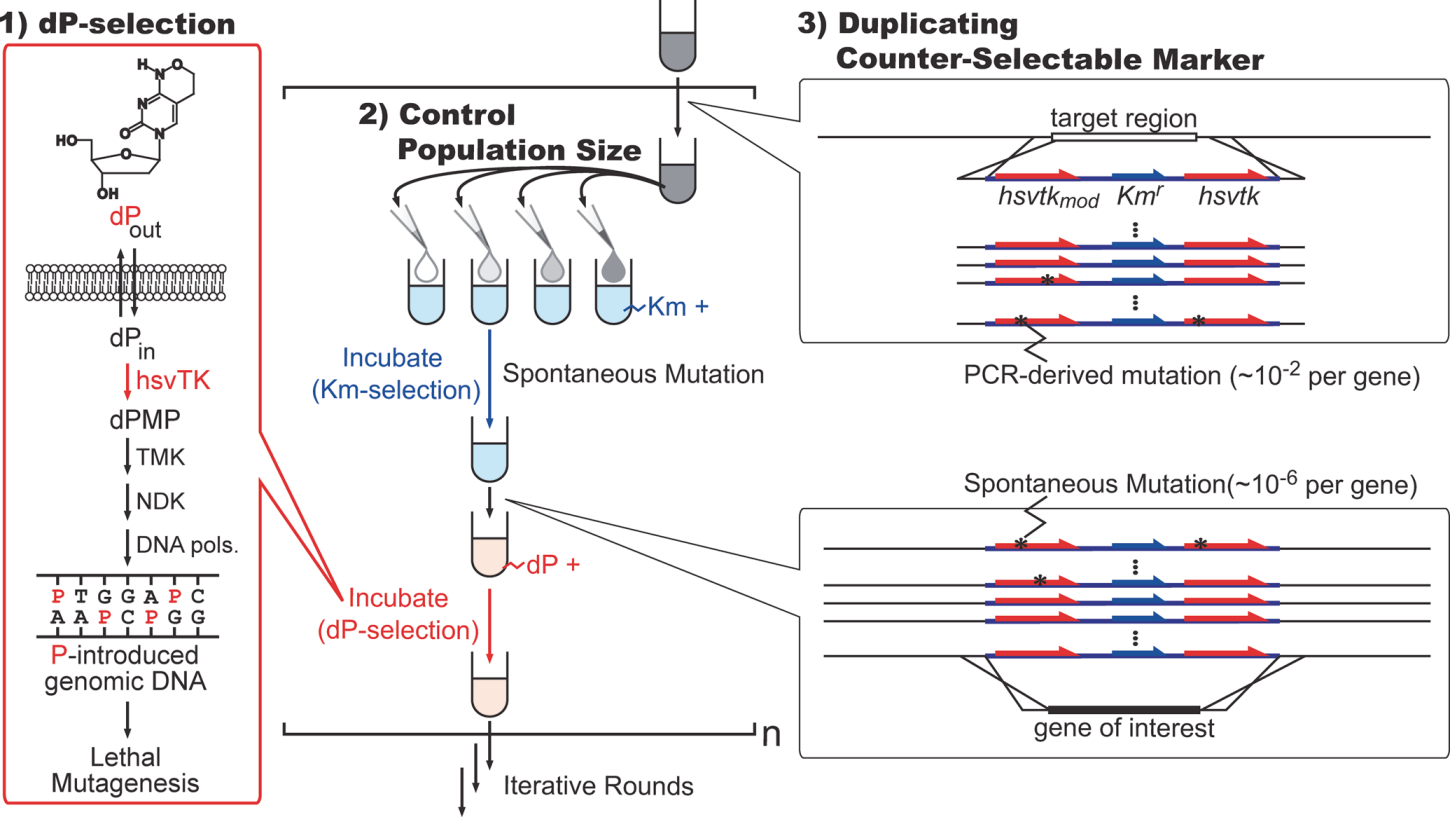
The recombineering protocol adapted for multiple rounds. First, the dP kinase activity of hsvTK is used for rapid and efficient counter-selection in liquid media. The mechanism of action of dP-selection is shown in the left panel. Second, the number of clones to be transferred between the steps is limited by diluting/aliquoting the transformant cultures, to prevent the propagation and subsequent domination of clones with PCR-generated inactivating mutations. Finally, the gene coding for *hsvtk* was duplicated to drastically reduce the emergence of selection escapes. TMK: thymidylate kinase, NDK: nucleoside diphosphate kinase, DNA pols: DNA polymerases, Km: kanamycin.

In this paper, we describe our efforts to establish a repeatable workflow in which all recombination/selection steps can be rapidly and seamlessly operated using only liquid handling (Fig. 1). First, we report that the promiscuous activity of herpes simplex virus Thymidine kinase (hsvTK) [21] towards the unnatural nucleoside dP [22] can be used as a highly efficient and rapid counter-selectable marker for genome editing. Second, we also demonstrate that by duplicating the *hsvtk* genes while controlling the number of recombinants carried to the next round, counter-selection escapes can be effectively virtually exterminated from the experiments. This enables the seamless operation of parallel and iterative rounds of gene replacements and insertions, without the need to isolate and/or confirm the recombinants. The entire process is conducted via liquid handling only, making it adaptable for full automation.

## Material and Methods

### Materials

The 6-(ß-D-2-deoxyribofuranosyl)-3,4-dihydro-8*H*-pyrimido[4,5-c][1,2]oxazin-7-one (dP) nucleosides [22] were purchased from Berry and Associates (Bishop Circle East, Dexter, MI; cat. PY7270). Oligonucleotides were synthesized by FASMAC Co., Ltd (Kanagawa, Japan). All other chemicals and media were of the highest grade available. Antibiotics were added to the growth medium as required at the following concentrations: 50 μg/mL carbenicillin (Carb), 30 μg/mL chloramphenicol (Cm), and 50 μg/mL kanamycin (Km).

### Strains and plasmids

All plasmids used in this study are shown in Table 1. *E*. *coli* strain K-12 MG1655 was used throughout this study, although *E*. *coli* strain XL10-Gold (Stratagene, La Jolla, CA, USA) was used for the plasmid construction. The plasmid pKD46 [1], which enables the L-arabinose-mediated induction of the Lambda RED recombination system, was transformed into MG1655 and its derivative cells prior to the genome editing.

**Table 1:** Plasmids used in this study.

Plasmid name	Genotype	Ori/marker	Source
pMW-*hsvtk-cat*	*P*_*lac*_-*hsvtk-cat*	pSC101/Km^r^	This study
pHK	*p*_*T5*_-*hsvtk*_*mod*_-*p*_*L*_-*Km* ^*r*^	ColE1/Amp^r^	This study
pHKH	*p*_*T5*_-*hsvtk*_*mod*_-*p*_*L*_-*Km* ^*r*^-*p*_*tet*_-*hsvtk*	ColE1/Amp^r^	This study
pUC-*p*_*L*_-*gfp* ^*mut3*.*1*^	*p*_*L*_-*gfp* ^*mut3*.*1*^	ColE1/Amp^r^	This study
pJ204-*p*_*T5*_-*mrfp*	*p*_*T5*_-*mrfp*	ColE1/Amp^r^	This study
pET23d-*mrfp*	*p*_*T7*_-*mrfp*	pBR322/Amp^r^	This study
pKD46	*p*_*BAD/AraC*_-Lambda RED_*γβα*_	pSC101/Amp^r^	[1]

MG1655-AI (MG1655Δ*araB*::*T7rnap-tetA*) was constructed by replacing its *araB* gene with a PCR-amplified portion of AraC/pBAD-T7RNAP from BL21-AI (Invitrogen Life Technologies, Carlsbad, CA). MG1655-*tdk* (MG1655Δ*tdk*::*tetRA*) was constructed by replacing its thymidine kinase gene (*tdk)* with a PCR-amplified tetracycline resistant gene cassette (*tetRA)*. MG1655Δ*lac*Z::*hsvtk-cat* was constructed as follows: the *hsvtk-cat* (where *cat* encodes chloramphenicol acetyltransferase gene), flanked by homology arms targeting the *lacZ* locus, was PCR-generated using the appropriate primers/template (S1 Table) using Vent_R_ DNA polymerase (New England Biolabs). The resultant *hsvtk-cat* cassette (HC cassette) was electroporated into MG1655 cells, and the transformant cells were enriched in media containing Cm. The resultant MG1655Δ*lacZ*::*hsvtk-cat* cells were checked for their dP sensitivity (dP kinase activity) by spotting onto an LB-agar plate containing 1 μM dP.

### Construction of the DNA cassettes

Selection cassettes and insertion cassettes were PCR-amplified with primers that added the appropriate homology to the target genomic regions. The primer pairs used to generate each DNA fragment are summarized in S1 Table. Unless otherwise noted, PCRs were performed with KOD DNA polymerase (TOYOBO, Osaka, Japan). PCR products were DpnI–treated and gel-purified to eliminate the template (plasmid or genomic DNA).

### Preparation and use of electroporation-competent cells

Electroporation-competent cells were prepared as described by Datsenko and Wanner [1]. Briefly, cells harboring pKD46 were grown in LB medium supplemented with 50 μg/mL Carb and 10 mM arabinose to induce the expression of Lambda RED enzymes. When the OD (λ = 600 nm) of the culture reached 0.4–1.0, the cells were placed on ice and washed twice with ice-cold water and 10% v/v glycerol, followed by resuspension in 20% v/v glycerol to make an electroporation-competent stock.

To the resulting electroporation-competent cells (40 μL), 100 ng of purified PCR fragments was added on ice. The mixture was then subjected to electroporation in 0.1-cm-gap cuvettes (Bio-Rad, Hercules CA) at 1.8 kV in a Gene Pulser electroporation apparatus (Bio-Rad, Hercules CA).

### dP-selection and efficiency evaluation

The efficiency of counter-selection using chromosome-encoded *hsvtk*/dP was evaluated by replacing genome-integrated HC cassette by PCR-amplified 3 kbp fragments containing the *lacZ* gene flanked by 1 kbp homology arms; 500 ng of purified PCR fragment was electroporated to 40 μL of electroporation-competent MG1655Δ*lacZ*::*hsvtk-cat* cells. The resultant transformant culture was resuspended in 1 mL of LB medium and shaken at 37°C for 3 h. The cured cells were then inoculated into fresh LB medium containing dP (1 μM). After shaking for 1–8 h at 37°C, a portion of the culture was plated on LB agar plates with 0.4% wt/v X-Gal and 0.1 mM IPTG and incubated at 37°C. At each time point, the ratio of recombinants/non-recombinants was determined by the number-ratio of blue/white colonies.

### Determination of the frequency of escape in dP-selection

Overnight cultures of the MG1655 strain or its derivatives were inoculated into fresh LB plate containing dP (at a final concentration of 0–10^3^ nM) and appropriate antibiotics. The colonies (if any) were allowed to grow on the plate for 12 h at 37°C. The frequency of dP-selection escape was calculated by:

[Number of colonies observed on 1 μM dP plates]/[Number of colonies on the plate without dP]

### Mutation frequency of *E*. *coli* strain MG1655 in dP-containing medium

Approximately 10^6^ cells of the MG1655 strain harboring pKD46 were inoculated into fresh LB media (2 mL) containing ampicillin (100 μg/mL). Then, dP (at a final concentration of 0–10^3^ nM) was added to the culture. After 6 hours of shaking, a portion (1 mL) of the culture was collected, quickly washed in fresh media, and then plated on LB-agar with or without rifampicin (rif assay [23]). The colonies (if any) were allowed to grow on the plate for 12 h at 37°C. According to the literature [24], there are ten unique nucleotide positions in *rpoB* that can alone confer resistance to rifanpicin resistance to *E*. *coli*. Given this, the mutation frequency of each sample was defined using the following equation. Mutation frequency [mutation/bp] = Rifampicin resistant clones [c. f. u.]/total cell number [c. f. u.] × 10

### Sequential operation of gene insertion/replacement

Step 1: The PCR-amplified selection cassette flanked by the appropriate homology arms (100 ng) was electroporated into electroporation-competent MG1655-AI cells (40 μL). The transformants were immediately resuspended in 1 mL of LB-Carb medium for at 30°C for 3 h. Usually the culture contained 10^1^–10^4^ recombinants depending on the homology-arm length and the length of DNA fragment. Thereafter, the culture was serially conducted 10-fold dilution and divided into aliquots in some of which the number of recombinants (Km-resistant cells) was limited to less than 10, where the probability that the aliquote contains false-positive clones are expected to be less than 2% (calculation provided in Discussion). More specifically, the aliquots was inoculated into fresh LB medium (2 mL) with Carb and Km and incubated for 24 h at 30°C. From the visibly turbid culture with highest dilution rate, cells were harvested and inoculated into fresh LB medium containing Carb, Km, and arabinose (10 mM) used for preparing electropotation-competent cell for recombineering as described above.

Step 2: A purified DNA fragment coding *mrfp* under the control of the T7 promoter and flanked by appropriate homology arms (*p*_*T7*_-*mrfp*, 100 ng) was added to the prepared electroporation-competent cells (40 μL). The resulting mixture was electroporated and resuspended in 1 mL of LB-Carb medium. After curation (3 h at 30°C), a portion of the culture was inoculated into fresh LB medium containing Carb and dP (1 μM). The aliquot was shaken at 30°C for 18–30 h, until it reached to stationary phase. Harvested cells were inoculated into fresh LB medium containing Carb and arabinose (10 mM) used for preparing electropotation-competent cell for recombineering as described above. This stock was then used in Step 1 of the next round.

Four rounds of recombineering (two-step replacement of the target sequences with *p*_*T7*_-*mrfp*) were conducted by repeating Step 1 and Step 2, targeting *yiiDE*, *proV*, *lacZ*, and *rssB* in a sequential manner.

### Fluorescence analysis

The fluorescence of the *E*. *coli* strains was measured with a Fluoroskan Ascent (Thermo-Labsystems, Helsinki, Finland). The following excitation/emission pairs (in nm) were used: GFP^mut3.1^, 485/527, mRFP, 584/620. To correct for variations in cell number, the fluorescence intensities were normalized by the cell density (OD_600_) of the culture measured in transparent 96-well plates with a SpectraMax Plus 384 system (Molecular Devices, Sunnyvale, CA).

Flow cytometric analyses of the cell mixtures, were conducted as follows: immediately after each round of recombineering (or after recovery from the glycerol stock), the cell mixture was grown in LB medium in test tubes (at 37°C at 200 rpm). Then, 20 μL of the overnight cultures was added to 2 mL of fresh LB medium containing 10 mM arabinose and shaken in a test tube at 37°C for 3 h. Approximately 10,000 cells were applied to a MACSQuant VYB flow cytometer (Miltenyi Biotech, Bergisch-Gladbach, Germany) equipped with a 561 nm laser and appropriate filter sets for mRFP (586/15). The data were analyzed by using the MACSQuant Analyzer (Miltenyi Biotech, Bergisch-Gladbach, Germany).

## Results

### Chromosomal insertion of *hsvtk*

HsvTK has long been used as a selectable marker in *E*. *coli* [25,26]. In this system, selection is performed by adding 2’-deoxy-5-fluorouridine (5FdU) to the culture media. Here, 5FdU was endogenously phosphorylated to 5FdU-MP, a potent inhibitor of thymidylate synthase (thyA). By blocking the *de novo* biosynthesis of dTMPs [25,26], cell growth is prevented. When dT is exogenously added and hsvTK is expressed, the biosynthesis of dTMPs (and cell growth) is restored via its salvage pathway.

We first attempted to apply this mechanism for the selection of the recombinants. We designed a selection cassette encoding hsvTK and another selectable marker chloramphenicol acetyltransferase (CAT) (HC cassette). Using the PCR primers shown in S1 Table, we generated double-stranded DNA that harbors HC cassette flanked with short homology arms (191 nt and 41 nt in length, respectively) targeted to the *lacZ* locus. The DNA fragment (2,132 bps) was electroporated into a cell harboring the Lambda RED system, followed by plating on Tryptone agar containing 5FdU/dT. Although this process successfully enriched the recombinants (MG1655-*tdk*, Δ*lacZ*::*hsvtk-cat*), they consisted only a part of the surviving population. We found the resulting recombinants selectively grew in media containing 5FdU/dT (data not shown), indicating that the titer of the thymidine salvage pathway is sufficiently high with single-copy *hsvtk*. However, we observed the frequent emergence of 5FdU-resistant clones, possibly because many different amino acid substitutions can confer thyA resistance to its inhibitors (5FdU) [27]. In contrast, we could easily isolate the right recombinants using chloramphenicol selection (Cm-selection) without being bothered by false-positive clones.

### dP kinase as a counter-selectable marker for genome engineering

We recently reported that hsvTK has efficient kinase activity against the nucleoside analogue dP [21]. When hsvTK is expressed from middle- to high-copy number plasmids, cells efficiently incorporate dP into the genomic DNA. Because P can base-pair with either A or G [22,28], its incorporation results in the destruction of the genetic information of the cell. By adding dP to the growth media, plasmid-borne *hsvtk* effectively and rapidly kills the host *E*. *coli* strain (#1 in Fig. 1). This system (dP-selection) was originally developed to select for the *OFF* state of the genetic switches and circuits on multicopy plasmids [21]. It has not been tested whether similar killing efficiency can be produced using chromosome-encoded *hsvtk*.

**Fig 2:**
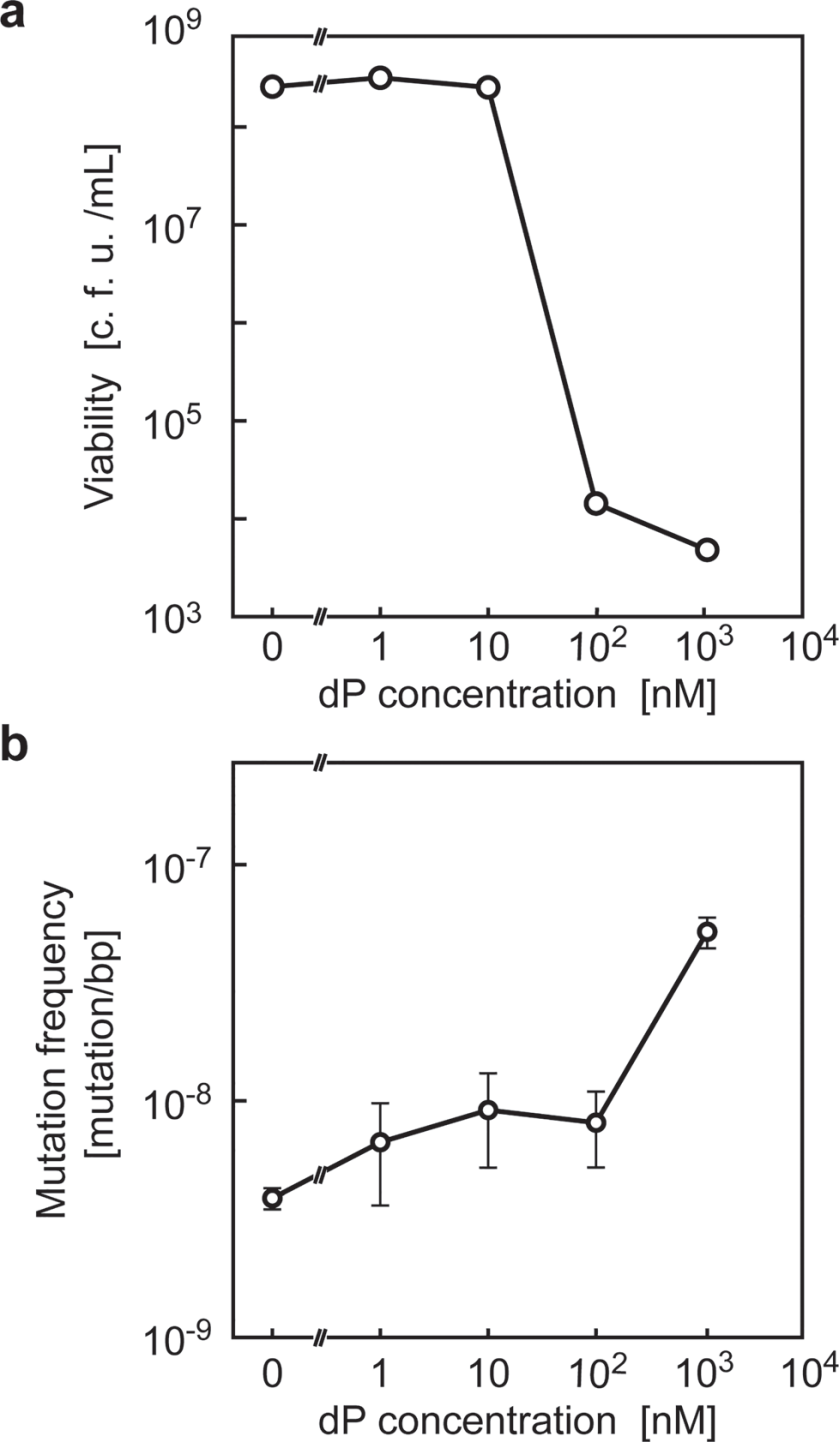
dP/hsvTK counter-selection. (a) Host-killing efficiency of dP-selection with different dP concentrations for *E*. *coli* strain MG1655Δ*lacZ*::*hsvtk-cat* harboring pKD46. (b) Mutation frequency of *E*. *coli* strain MG1655/pKD46 in the media containing varying concentration of dP. The bar heights show the average of 3 samples, and error bars indicate the standard deviation.

HC cassette was electroporated to MG1655, followed by Cm-selection. From the resultant transformants (MG1655-*tdk*Δ*lacZ*::*hsvtk-cat*), we randomly picked 92 clones and tested for their dP kinase activity. We found that most (89/92) of the clones completely lost their cell viability (not shown), indicating that the activity of chromosome-coded *hsvtk* is still high enough to cause cell death of the hosts, making it useful for genome engineering. When tried-and-true dP-sensitive clone (MG1655Δ*lacZ*::*hsvtk-cat*) was tested on dP-selection plate containing 1 μM dP, 5 × 10^3^ escape colonies were appeared per 3 × 10^8^ colonies on LB plate without dP, thereby yielding selection escape frequency of 2 × 10^−5^ (Fig. 2A). With this culture condition, the mutation frequency of the cells to be selected (those not expressing hsvTK) was only 10-fold higher than untreated cells (Fig. 2B).

### Time required for dP-selection

**Fig 3:**
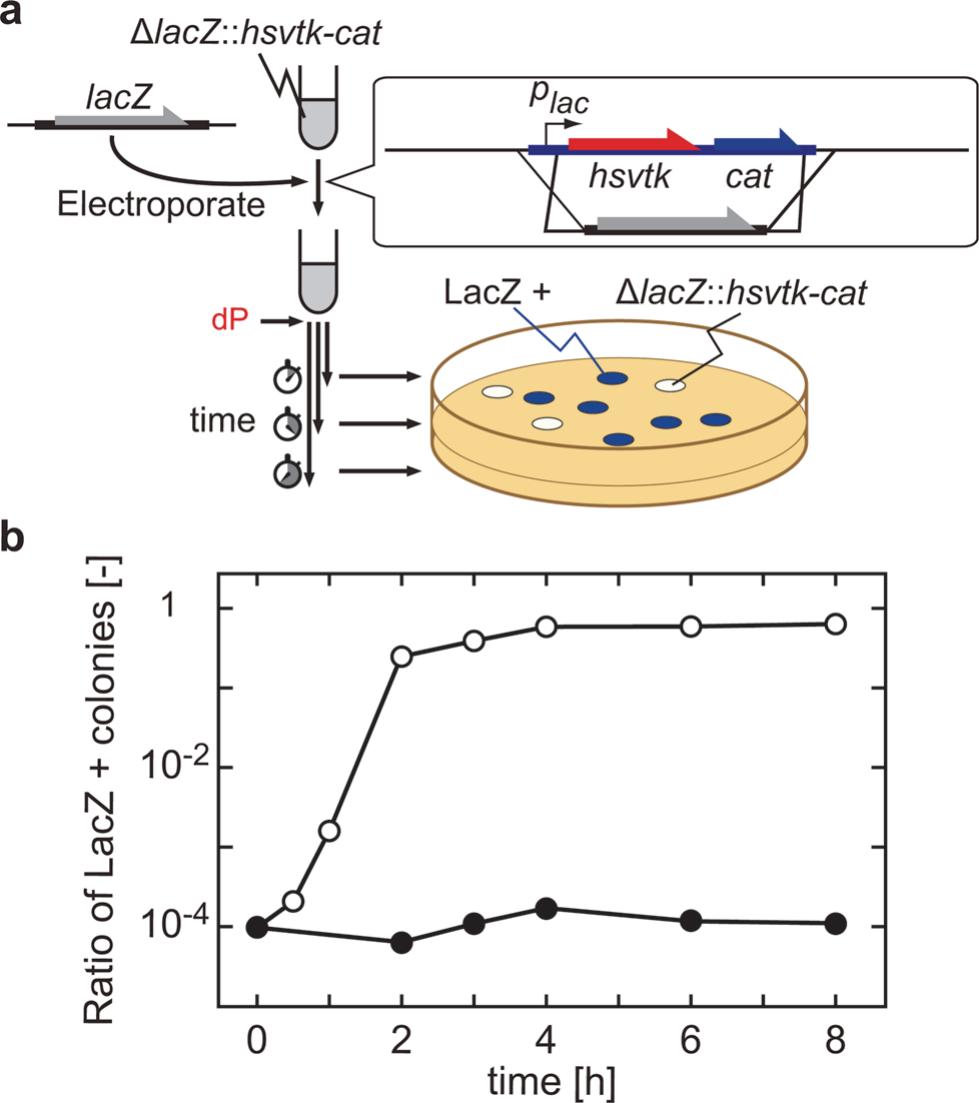
Liquid-based counter-selection using dP kinase activity. (a) Experimental procedure. The selection cassette inserted in *lacZ* region of *E*. *coli* chromosome was replaced back to *lacZ* by eletroporating PCR-generated *lacZ* gene. Transformant cells were incubated at 37°C in dP-containing LB medium, and a portion of the culture were plated at each time points (0–8 hrs). The number of recombinants was estimated by counting the number of blue colonies per plate. (b) Ratio of recombinant cells (LacZ +) various periods after adding dP, with (open circles)/without (closed circles) dP treatment (1 μM).

Another unique feature of the dP-selection is the speed of the selection procedure [21]: on plasmid, selection time could be reduced to as short as 5 min. To determine whether this is also the case in genome engineering, we analyzed the effect of the selection time on the selection efficiency. We attempted to replace the HC cassette in the chromosome of MG1655Δ*lacZ*::*hsvtk-cat* cells with *lacZ* (Fig. 3A). After electroporating a PCR-generated *lacZ* gene, the transformant culture was split into two and then shaken in the presence or absence of dP (1 μM). A portion of the culture was collected at various time points and plated onto LB plates containing X-gal. At time zero, the amount of LacZ positive (blue) clones was only 10^−4^ (*ca*. 100 recombinants/10^6^ total cells/mL). In the culture without dP, this rate was unchanged during the experiment. In the culture containing dP, this fraction drastically increased over time (Fig. 3B). Because dP-based counter-selection sterilize cells rather than inhibit cell growth [21], this selection does not require overnight growth. Indeed, the selection efficiency quickly plateaued approximately 2 hours after dP addition.

### Inactivation of counter-selectable markers by PCR errors

During the creation of MG1655-*tdk*Δ*lacZ*::*hsvtk-cat*, three dP-resistant clones emerged (see above). We found that all three clones possessed non-synonymous and likely inactivating mutations within the coding regions of *hsvtk* (S2 Table). These mutations were likely derived from replication errors in PCR amplification: we found that the frequency of dP-resistant/Cm-resistant clones (which possess HC cassette with an inactivated *hsvTK*) is dependent on the fidelity of the polymerase used for the PCR amplification of the cassette (S3 Table). Postulating that 25% of nucleotide substitutions result in the deleterious missense/nonsense mutations [29,30], and given the inactive fraction of *hsvtk* to be 0.8% (S3 Table), nucleotide mutations per *hsvtk* gene can be calculated to 0.8%/0.25 = 3.2% for HC cassette amplified with Vent_R_ DNA polymerase. Given the nucleotide size of *hsvtk* (1,129 bps) and amplification factor 10, corresponding to ca. 1,000 fold amplification, error rate of Vent_R_ DNA polymerase is calculated to be 0.032 [mutation/gene]/10 [amplification factor]/1,129 [bps/gene] = 3 × 10^−6^. These values are similar to the reported error values for Vent_R_ DNA polymerase (2.8 × 10^−6^ mutations/base/replication [31]). Thus, inactivating mutations are not enriched in *hsvtk*, indicating that *hsvtk* is not under strong counter-selection in *E*. *coli*. This is in contrast to other counter-selectable markers such as SacB, which is known to be under severe counter-selection [32,33]. Note that the native (cognate) function of hsvTK (dT kinase) is non-toxic; its toxicity is exerted only when dP is added to the media.

### False-positive clones generated by errors in chromosomal replication

Bacterial chromosomes are continuously accumulating spontaneous mutations over generations, such that any given gene could be inactivated with a certain frequency. Even starting from tried-and-true dP sensitive clone, we obtained one dP-resistant clone for every 10^5^ cells after the selection step (Fig. 2A). Given 10^−5^ as then inactive fraction of *hsvtk* (Fig. 2A) and postulating about 25% of nucleotide substitutions are inactivating [29,30], and nucleotide mutations per *hsvtk* gene can be calculated to 10^−5^/0.25 = 4 × 10^−5^. Considering that the cell pool was grown for 20 generations (10^6^ fold amplification), error rate of chromosome replication is calculated to be 4 × 10^−5^ [mutation/gene]/20 [amplification factor]/1,129 [bps/gene] = 2 × 10^−9^, which is close to the reported values (10^−10–^10^−9^ [34]). Considering the number of recombinants (10^−5^–10^−4^ with the aid of Lambda RED system) that we routinely obtain, dP-resistant clones (10^−5^) generated by errors in chromosomal replication represent a non-negligible source of false-positives.

### Mutational escape impedes the continuous operation of genome engineering

**Fig 4:**
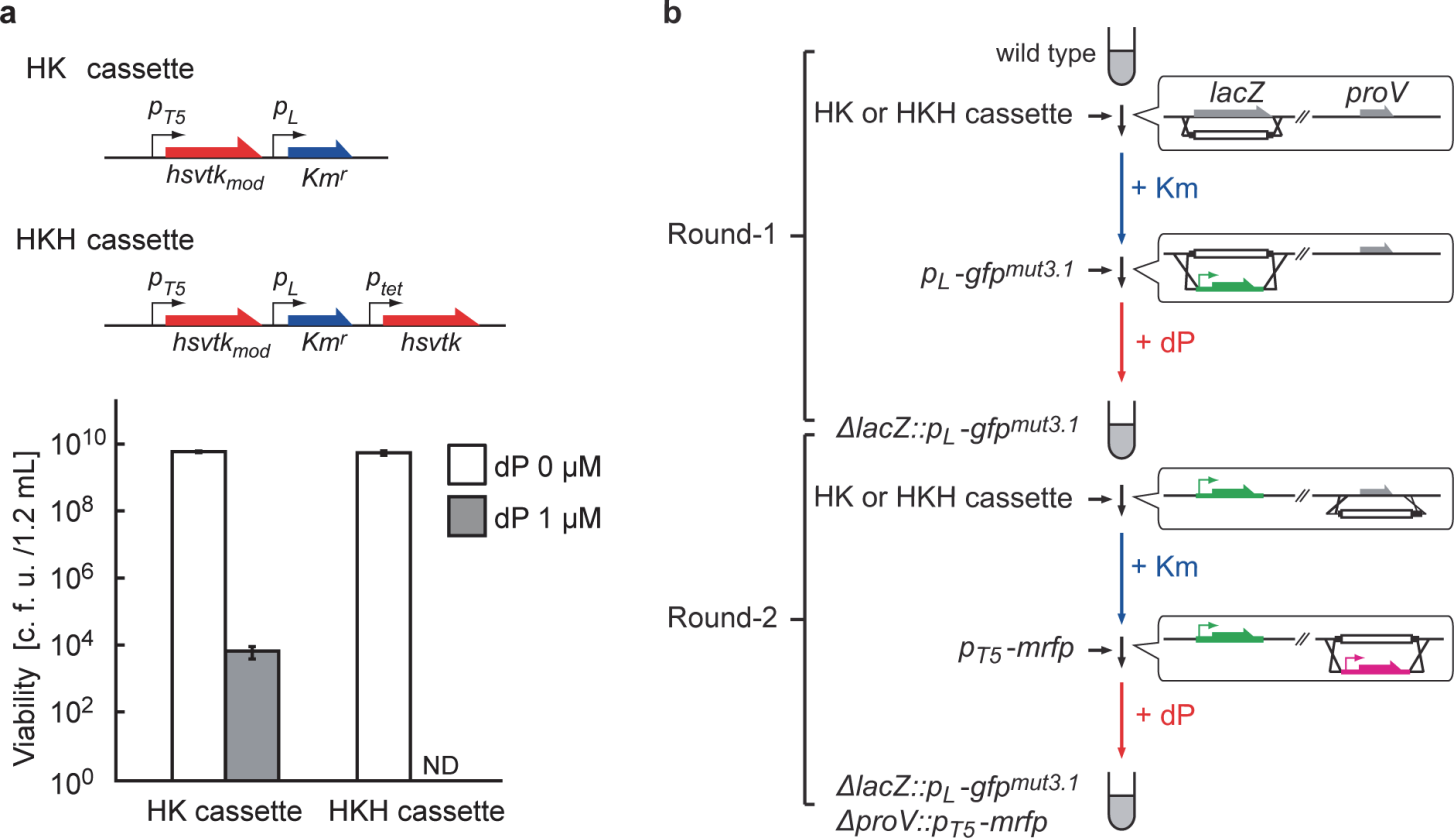
Duplication of *hsvtk* enables a two-round consecutive recombineering. (a) Killing efficiency of dP-selection against *E*. *coli* strain harboring a single *hsvtk* gene (HK cassette) or duplicated *hsvtk* (HKH cassette), ND: Not detected. The bar heights show the average of 3 samples, and error bars indicate the standard deviation. (b) The experimental workflow for a two-round consecutive recombineering: *lacZ* and *proV* loci were serially replaced with *pL-gfp* ^*mut3*.*1*^ and *pT5-mrfp* cassette respectively using HK or HKH. All DNA cassettes used here were prepared with 40-bp homology arms to the individual target sites, except the step for replacing *lacZ* with *pL-gfp* ^*mut3*.*1*^ cassette where 1-kbp homology arms were adopted.

Once chromosomal *hsvtk* is inactivated by mutations, either by PCR error or by cellular spontaneous mutations, the resultant clone is expected to quickly dominate the population at the subsequent step (dP-selection). To confirm this, we attempted the two gene replacement steps (Δ*lacZ*::*p*_*L*_-*gfp* ^*mut3*.*1*^ and Δ*proV*::*p*_*T5*_-*mrfp*) by using *hsvtk-Km* ^*r*^ cassette (where *Km* ^*r*^ encodes a kanamycin resistant gene, so called ‘HK cassette’) (Fig. 4).

We conducted the first round of recombineering, where we inserted *p*_*L*_-*gfp* ^*mut3*.*1*^ [35] via a deletion coupled insertion of HK cassette, resulting in MG1655Δ*lacZ*::*pL-gfp* ^*mut3*.*1*^. After this round, 90% (85 of 94 clones) of the resultant population showed fluorescence of GFP^mut3.1^, whereas the rest of the populations (9 clones or 10% of the entire population) did not (Table 2). Next, we pooled all of the obtained clones without removing these false-positive clones and proceeded to the second round of recombineering (Δ*proV*::*p*_*T5*_-*mrfp*). In this round, we obtained numerous non-fluorescent colonies after the first-half step (insertion of the HK cassette followed by kanamycin selection (Km-selection)). After the completion of the procedure for the second-half step (electroporation of the *p*_*T5*_-*mrfp* cassette followed by dP-selection), the pool was completely dominated by non-fluorescent clones with the Km^r^ phenotype (Table 2). We picked nine of the non-fluorescent clones in the pool after the first round and sequenced their Δ*lacZ*::*hsvtk-Km* ^*r*^ locus (S4 Table). The eight out of nine non-fluorescent clones were selection-escape clones retaining HK cassettes with mutations in *hsvtk*: four had non-synonymous mutations in the *hsvtk* gene, three had a deletion of the entire *hsvtk* gene, and one was lacking the entire HK cassette. We found one non-fluorescent clone that had the *gfp* ^*mut3*.*1*^ gene at the proper position but also contained a stop codon in the same reading frame. We do not know where these mutants were generated. Note that we did not select for the acquisition of fluorescence.

**Table 2:** Functional and genotype analysis of recombinant pools.

		Round-1			Round-2			
Phenotype (fluorescence)	GFP	+	-		+	+	-	+
	RFP				+	-	+	-
genotype^1^ (PCR)		NT^2^	+	-	NT^2^	+	+	NT^2^
HK cassette		85(90%)	1(1%)	8(9%)	0(0%)	0(0%)	0(0%)	94(100%)
HKH cassette		94(100%)	0(0%)	0(0%)	90(96%)	2(2%)	2(2%)	0(0%)

After each round of recombineering depicted in Fig. 4B, 94 clones were randomly picked, grown, and analyzed with regard to both phenotype (fluorescence) and in genotype (genomic PCR).

^1.^ Number of clones with PCR bands corresponding to Δ*lacZ*:: *p*_*L*_-*gfp* ^*mut3*.*1*^ (for round-1) or Δ*lacZ*::*p*_*L*_-*gfp* ^*mut3*.*1*^ and Δ*proV*::*p*_*T5*_-*mrfp* (for round-2). For the sequences of primers used for genotype analysis (P34 and P42 for *p*_*L*_-*gfp* ^*mut3*.*1*^, P35 and P39 for *p*_*T5*_-*mrfp*), see S1 Table.

^2.^ NT; not tested.

### Duplication of the counter-selectable marker

A drastic decrease in the spontaneous mutation rate is known to be difficult to achieve [36]. The inactivation of the *hsvtk* gene is therefore an inevitable event in a cell population. We designed a new selection cassette that contains two copies of the *hsvtk* gene. The idea was that dP kinase activity should be maintained unless both of the *hsvtk* genes are inactivated. Because the frequency of *hsvtk* gene to be inactivated is 10^−6^/cell for HK cassette (Fig. 4A), the frequency of inactivating both *hsvtk* genes should theoretically be 10^−12^/cell/step (#3 in Fig. 1). Considering the number of transformants (10^−5^–10^−4^/round [2] with 40-base homology arms) that we routinely obtain, the fraction of escapee is estimated to be as low as 10^−8^–10^−7^ of the population.

We constructed a new selection cassette with two *hsvtk* genes (HKH cassette, Fig. 4A). Here, it was expected that simple duplication in *hsvtk* would result in highly frequent homologous recombination between these genes, thereby promoting the appearance of dP-resistant clones. In *E*. *coli*, homologous recombination occurs most frequently when there is a contiguous stretch of sequence >25 bp [37–39]. We therefore synthesized the codon-modified *hsvtk* (*hsvtk*_*mod*_), which has 72% nucleotide identity to the original version. In this design, the longest stretch of successive nucleotides identical to the original *hsvtk* contained eleven nucleotides (S1 Fig.). Also, we placed the selectable marker (*Km* ^*r*^) between the two *hsvtk* genes. Thus, if inter-*hsvtk* recombination occurred, it would result in the loss of Km-resistance as well, thereby effectively removing the clone in subsequent rounds of genome engineering. When *E*. *coli* strain MG1655Δ*lacZ*::*hsvtk*_*mod*_-*Km* ^*r*^-*hsvtk*) was tested on dP-selection plate (containing 1 μM dP), no counter-selection escape colonies were appeared per 6 × 10^9^ colonies on LB plate (without dP) (Fig. 4A).

We conducted continuous two-step recombineering with this new HKH cassette (Fig. 4B). This time, all 94 tested clones were fluorescent and Km-sensitive after the first round of Km-/dP-selection (Table 2). This indicates that the cell population was virtually free from selection escape (clones holding a mutated HKH cassette). The cell mixture was directly subjected to the second round of recombination. Even after the second round of recombineering (Δ*proV*::*p*_*T5*_-*mrfp*), all (94/94) of the tested clones were double-recombinants (harboring both *p*_*L*_-*gfp* ^*mut3*.*1*^ and *p*_*T5*_-*mrfp* cassettes) (Table 2). Thus, duplicating the counter-selectable marker gene (*hsvtk*) significantly reduced the counter-selection escape, allowing us to directly apply the recombinant pool to subsequent rounds of recombineering without colony isolation or genotyping.

### Continuous multiple gene replacements with mRFP

**Fig 5:**
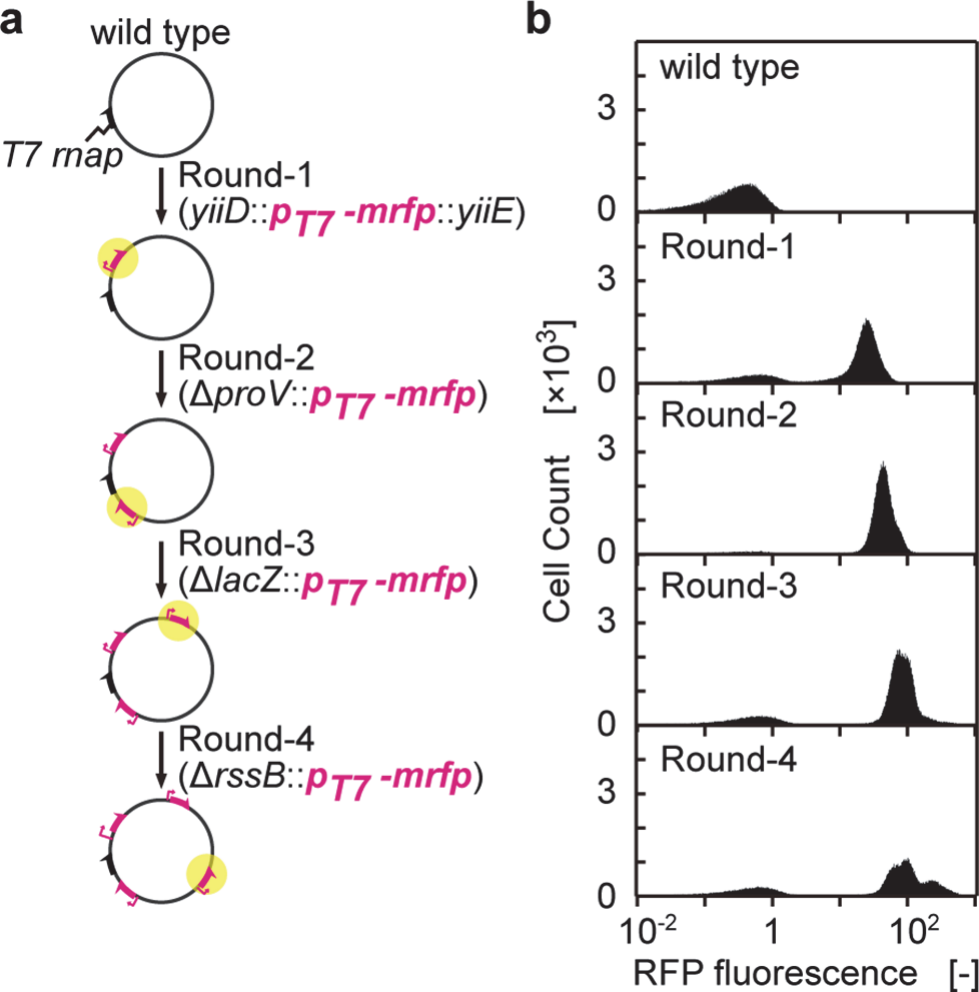
Sequential replacement of four chromosomal loci with DNA fragments encoding *p*_T7_-*mrfp*. (a) The production flow chart for four-round gene replacement. (b) Flow cytometric analysis of the recombinant pool after each round of recombineering.

Having established a seamless workflow for multi-round selection-based recombineering (Fig. 4B), we attempted to apply the method for the iterative replacement of four selected loci, *yiiDE*, *proV*, *lacZ*, and *rssB*, to an *E*. *coli* chromosome with a PCR-generated fragment harboring *mrfp* under the T7 promoter (*p*_*T7*_-*mrfp*) (Fig. 5A). To enable the expression of mRFP, *araB* locus of MG1655 was replaced with T7 *rnap*. The resultant strain MG1655-AI was subjected to the four-round recombineering. In each round, the PCR-amplified HKH cassette with homology arms (40 bp in length) targeted to the corresponding sites was electroporated into the cells (MG1655-AI), followed by Km-selection (24 h). *p*_*T7*-_ *mrfp* was electroporated into the resultant cell culture, followed by 18–30 hours of growth in media containing dP. The resultant culture (recombinant pool) was divided into two pools: one was stored as an intermediate recombineering pool for analysis, whereas the other pool was subjected to the next round of recombineering. This procedure was repeated without break for four rounds.

At each step of the four-round gene insertions/replacements, we checked the phenotype (RFP fluorescence) of the recombinant pool via flow cytometry (Fig. 5B). The fluorescence signal progressively increased with increasing round number, indicating a step-by-step increase in the copy number of the *p*_*T7*_-*mrfp* cassette on the chromosome.

**Fig 6:**
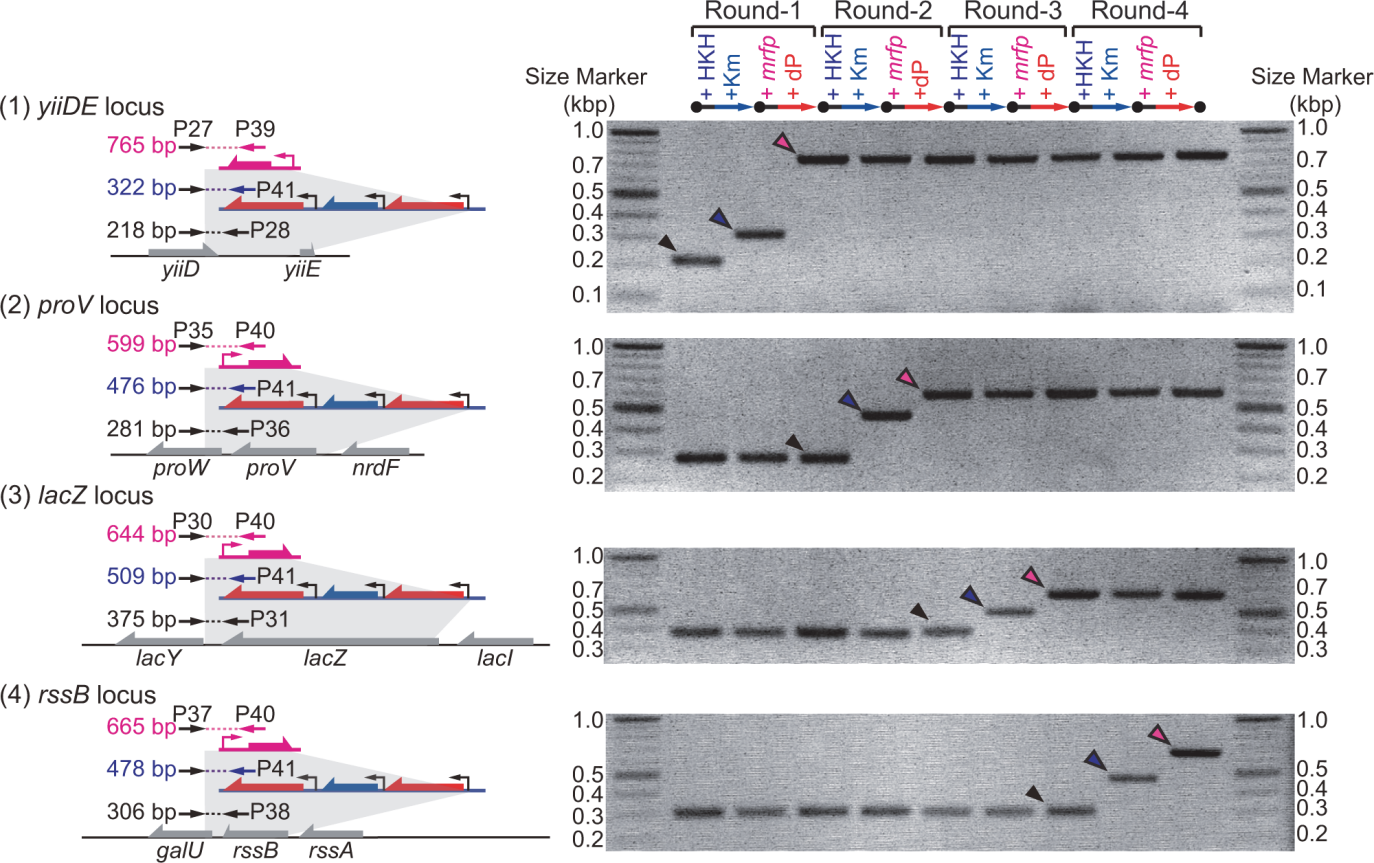
Genotyping of the cell population in each step of *mrfp* replacement in four different loci. Before, after, and during the 4 successive rounds of gene replacement, all 9 cultures were subjected to competitive PCR analysis using 3 primers annealing to the target, HKH cassette, and *p*_*T7*_-*mrfp* cassette sequences. The local sequence, location of the primers and expected size (in bp) of the PCR products are presented for the (1) *yiiDE*, (2) *proV*, (3) *lacZ*, and (4) *rssB* loci. The sequences of the primers used are shown in S1 Table.

The progressive integration of the *p*_*T7*_-*mrfp* cassettes was also verified by PCR analysis (Fig. 6). After each round of Km- or dP-selection, a portion of the ‘intermediate’ cell mixture was collected and subjected to PCR analysis. In each PCR analysis, a mixture of the four relevant primers (S1 Table) was used for each locus. The primer mix included the common forward primer and three reverse primers designed to anneal to the original target (non-recombinant), HKH cassette, or *p*_*T7*_-*mrfp* (see the left panel of Fig. 6). In the first round, only the band (218 bp) corresponding to the original sequences (*yiiDE*) was observable for the starting cell mixture. After the replacement of the sequence with the HKH cassette, we observed a single-banded PCR product (322 bp) corresponding to the HKH cassette inserted. After electroporation of the *p*_*T7*_-*mrfp* cassette followed by dP-selection, the band (765 bp) corresponding to the *p*_*T7*_-*mrfp*-inserted genome was observed as a single band. For each of all subsequent three rounds, we observed the complete shift in genotype at the targeted locus (Fig. 6). It should be noted that PCR was conducted directly from the culture, not from individual isolates. Thus, the data shown in Fig. 6 represent the genotype distribution of the entire population. No incorrect bands were detected in any of the eight steps (4 rounds), indicating that the number of clones with incorrect genotypes, including selection escape, was negligible. Indeed, we picked and tested 7 individual clones from the round-4 cell population (S2 Fig.) and could not identify a single clone that gives the incorrect PCR patterns.

We have not invested any effort in shortening the operation time of each step. Nevertheless, one round is completed in two to three days (see the experimental section), depending on the growth rate of the recombinants. In this case (Figs. 5 and 6), four rounds of recombineering were completed in ∼10 days. Also note that all of the steps were conducted using only liquid handling, without the need for a manual procedure for identifying the right recombinants by colony picking and conducting multiple PCR reactions for each colony.

The cellular fluorescence slowly leveled off with the increase in recombineering rounds. Additionally, we observed a significant retardation in the growth rate of cells in later rounds. We do not know whether this retardation came from: it could be possibly ascribed to the metabolic load of over-expression of RFP, to the multiple deletions of four genes, or by accumulated mutations throughout the chromosome. At the fourth round, we observed a two-peaked fluorescence, together with a minor fraction with no fluorescence. We believe that the lower peak of the two-peaked fluorescence represents clones with three functional *mrfp* and one *mrfp* inactivated by PCR errors, whereas the non-fluorescent peaks represent clones with inactivation mutations in the reading frame of the T7 RNA polymerase.

## Discussion

Rapid advances in the field of synthetic biology are creating a demand for robust, broadly accessible methodologies for manipulating multiple genomic loci in a high-throughput manner. In this study, we described dP kinase activity can be used as highly efficient and rapid counter-selectable marker in genome engineering (Figs. 2 and 3). Because all required procedures can be completed by liquid handling only, different recombineering projects can be conducted in parallel and in multi-well formats.

Our dP-selection is mechanistically based on the lethal mutagenesis [28], which is not generally favored by genome engineers. However, with our protocol (*i*.*e*. treatment with 1,000 nM dP), the mutation frequency of the cells to be selected (those not expressing hsvTK) was only 10-fold higher than that of cells not treated with dP (Fig. 2B). Note that this mutation frequency is much lower than that of the *mutS* mutant strain (100-fold higher mutation frequency than that of wild type strain [40]) used in the MAGE system. We found no detectable increase in the chromosomal mutation frequency of *E*. *coli* in the presence of 100 nM dP. Engineering or searching for a more efficient dP kinase (with a lower K_M_ for dP) or over-expression of nucleoside importers [41] could be effective in further decreasing the concentration of dP required for the counter-selection process, thereby also decreasing the mutation rate of the recombinant cells.

The largest obstacle for the continuous/automatable operation of gene-size recombineering is the emergence and propagation of selection escapes generated by inactivating mutations in counter-selectable marker genes. By duplicating the *hsvtk* genes (using the HKH cassette), the frequency of their appearance could be reduced to <2 × 10^−10^/cell during cell growth (Fig. 4A). We picked nine dP-resistant clones and sequenced in its *hsvtk* locus. All clones had mutations in *hsvtk* (S5 Table). We confirmed that these mutations alone confer dP-resistance to *E*. *coli* (S3 Fig.). These results indicate that, very few, if not zero, of the escape events comes from the off-site mutations in dP-selection. This is in sharp contrast to other counter-selection mechanisms: for example, Gregg *et al*. reported that in tolC-based systems, ‘off-target’ inactivating mutations accounted for significant part of the escape events [11]. They succeeded in lowering the frequency of selection escape not by duplicating the selector *tolC* cassette, but rather by duplicating the off-site mutational hotspot (*tolQRA)* [11]. Or, selection stringency could be improved by combinatorial use of different types of counter-selectable markers in tandem to improve the selection stringency [5,6,11].

Although duplication of *hsvtk* was highly effective in decreasing the frequency of the dP-resistant cassette, we still observed a non-negligible frequency of clones with the dP-resistant cassette. When the HKH cassette was PCR-generated, where 0.2–0.8% of individual *hsvtk* could receive inactivating mutation (S3 Table), up to 0.4–6 × 10^−5^ of the genome-integrated HKH cassette could lack dP kinase activity. This is comparable to the rate of recombination (10^−5^ to 10^−4^/cell [2]).

The appearance of such parasitic entities has also been an important issue for autonomous replication systems [42–44]. Once they appear, they can eventually dominate the population and inhibit the further evolution of replication systems. Theoretical [45–47] and experimental [44] works have shown that spatial structures such as compartments could effectively repress the propagation of parasites by quarantining them and thereby preventing them from taking over the entire population.

Inspired by this, we split the pool of recombinants and restricted the number of recombinants in each pool to be fed to the next step to be << 100 (#2 in Fig. 1). Throughout this work, we used the PCR-generated cassette to achieve fast-track genome engineering. Even with the highest-fidelity polymerases, 0.2% of the recombinants had inactivated *hsvtk* (S3 Table). When the number of recombinants per aliquot is set to be 10, the probability of having selection escape would be 2% and 0.004% for HK cassette and HKH cassette, respectively. Although we have not encountered such throughout this work, each aliquot has a certain risk to be contaminated with selection escapes generated by PCR error, and the risk increases with the increase of the pool size. Because recombination efficiency is context dependent *per se*, the proper dilution ratio could differ every single time. Our recommendation is to prepare the dilution series of the recombinant pool such that several dilutions can be independently subjected to the next round of recombineering in parallel: the contaminated aliquot, if appears, could be easily distinguished from others in the subsequent processes. This way, the passage can be kept free from selection escapes, despite the lower replication fidelity of PCR.

Because PCR-error is much higher compared with genome replication [31], this removal of PCR-generated selection escape is the key. Once PCR-generated selection escapes are removed this way, or if the selection cassettes were directly prepared from sequence-confirmed plasmids, selection escapes (dP-resistant/Km-resistant clones) could be as low as 10^−6^ and 10^−12^ for HK cassette and HKH cassette respectively. If all DNA cassettes are prepared from plasmids (PCR-free), population-control procedure (#2 in Fig. 1) could be omitted.

In summary, we have established, for the first time, a repeatable workflow for gene replacement/insertion where all the steps can be seamlessly operated using only liquid handling with a re-designed selection cassette. Given its simplicity and flexibility, we predict that this proven technology will be valuable as a tool for high-throughput and multi-target genome editing projects.

## Supporting Information

S1 Fig Sequence alignment of wild-type *hsvtk* and its modified duplicate. Identical bases are highlighted with grey-shading.
(TIF)

S2 Fig Genotyping of four different loci of isolated clones from the round-4 cell population (Figs. 5 and 6). Seven clones were isolated and individually subjected to PCR analysis using primers annealing to the *mrfp* gene. The local sequence, location of the primers, and expected size (in bp) of the PCR products are shown for the (1) *yiiDE*, (2) *proV*, (3) *lacZ*, and (4) *rssB* loci. The sequences of the primers used are shown in S1 Table.
(TIF)

S3 Fig Evaluation of the dP sensitivity of MG1655 transplanted with on-site mutations identified in selection escapees. Mutant *hsvtk* gene identified in dP-selection escapees (S5 Table) were PCR-amplified and inserted into the *lacZ* locus of MG1655. The resultant recombinants were grown in LB media (0.5 mL) containing Km (50 μg/mL). From each culture, defined number of cells were plated onto LB-Km-agar containing 0 (open bar) or 1 μM (closed bar) of dP. Viability was determined by the colony forming units on each plate. The corresponding clone number in S5 Table is/are also given in parenthesis.
(TIF)

S1 Table List of primers used for the experiments in this work. (PDF)

S2 Table Sequence analysis of the *hsvtk* gene from dP-resistant clones that arose after the first step of recombination. Out of the 92 transformants (chloramphenicol-resistant clones isolated from the pool of MG1655 electroporated with HC cassette), we found three dP-resistant clones. The cassette inserted on chromosome was PCR-amplified using primer P30 and P32 (S1 Table) for the sequence analysis.
(PDF)

S3 Table Frequency of dP-resistant clones in MG1655Δ*lacZ*::*hsvtk-cat* created by HC cassette PCR-amplified using different polymerases. The HC cassette was amplified with either Vent_R_ DNA polymerase or Phusion DNA polymerase using primer P1 and P2 (S1 Table) to attach homology arms for targeting to the *lacZ* locus. The resultant DNA fragment was electroporated into *E*.*coli* strain MG1655 harboring pKD46 and plated onto an LB-Cm and LB-Cm/dP plate. The frequency of dP resistance (%) was calculated by: 100 × [number of colonies observed on Cm/dP plates] / [number of colonies on the Cm plate] The numbers show the average of 3 samples with standard deviations.
(PDF)

S4 Table Genotype summary of non-fluorescent clones found in the first round of recombineering. From the first round of two-step recombination using the HK cassette (Table 2), nine clones without GFP^mut3.1^ fluorescence were individually picked and analyzed in their *lacZ* (or Δ*lacZ*::*hsvTK-km* ^*r*^) locus. For the sequence of primers used for PCR amplification and sequencing (primers P30, 33, P43, and P44), see S1 Table.
(PDF)

S5 Table Mutations found in *hsvtk* regions of the counter-selection escapees arose during the overnight growth of a dP sensitive strain MG1655Δ*lacZ*::*hsvTK-km* ^r^. dP sensitive clone was cultured overnight and plated on dP-selection plates. From these plates nine clones were picked and sequenced in their *hsvtk* coding regions.
(PDF)
