# Supplementary figures and images for: Liquid-Based Iterative Recombineering Method Tolerant to Counter-Selection Escapes
Source: PLoS One. 2015 Mar 16;10(3):e0119818. doi: 10.1371/journal.pone.0119818 (PMC4361647; doi:10.1371/journal.pone.0119818)

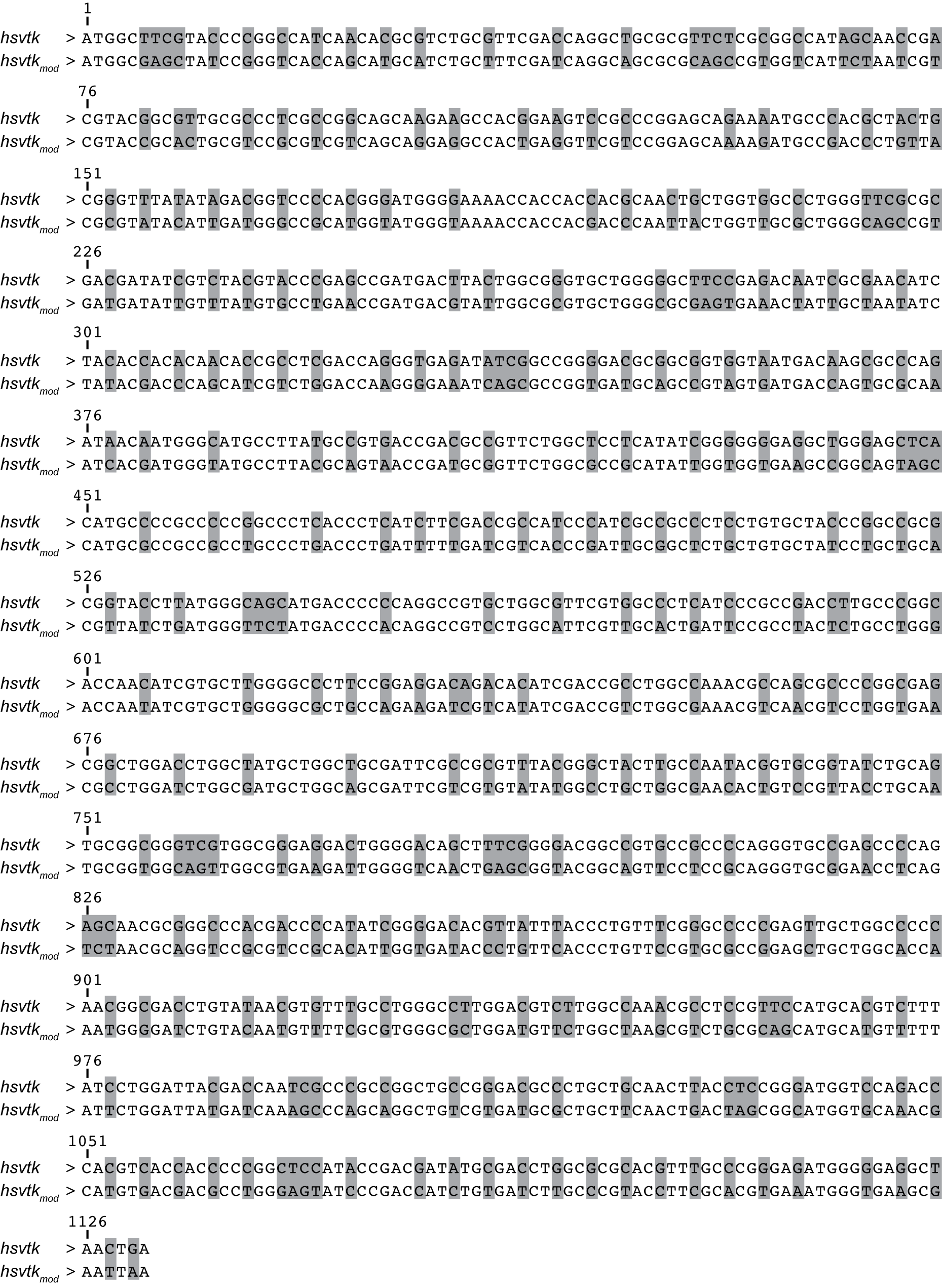

Supplement: S1 Fig — Identical bases are highlighted with grey-shading. (TIF) [file pone.0119818.s001.tif]

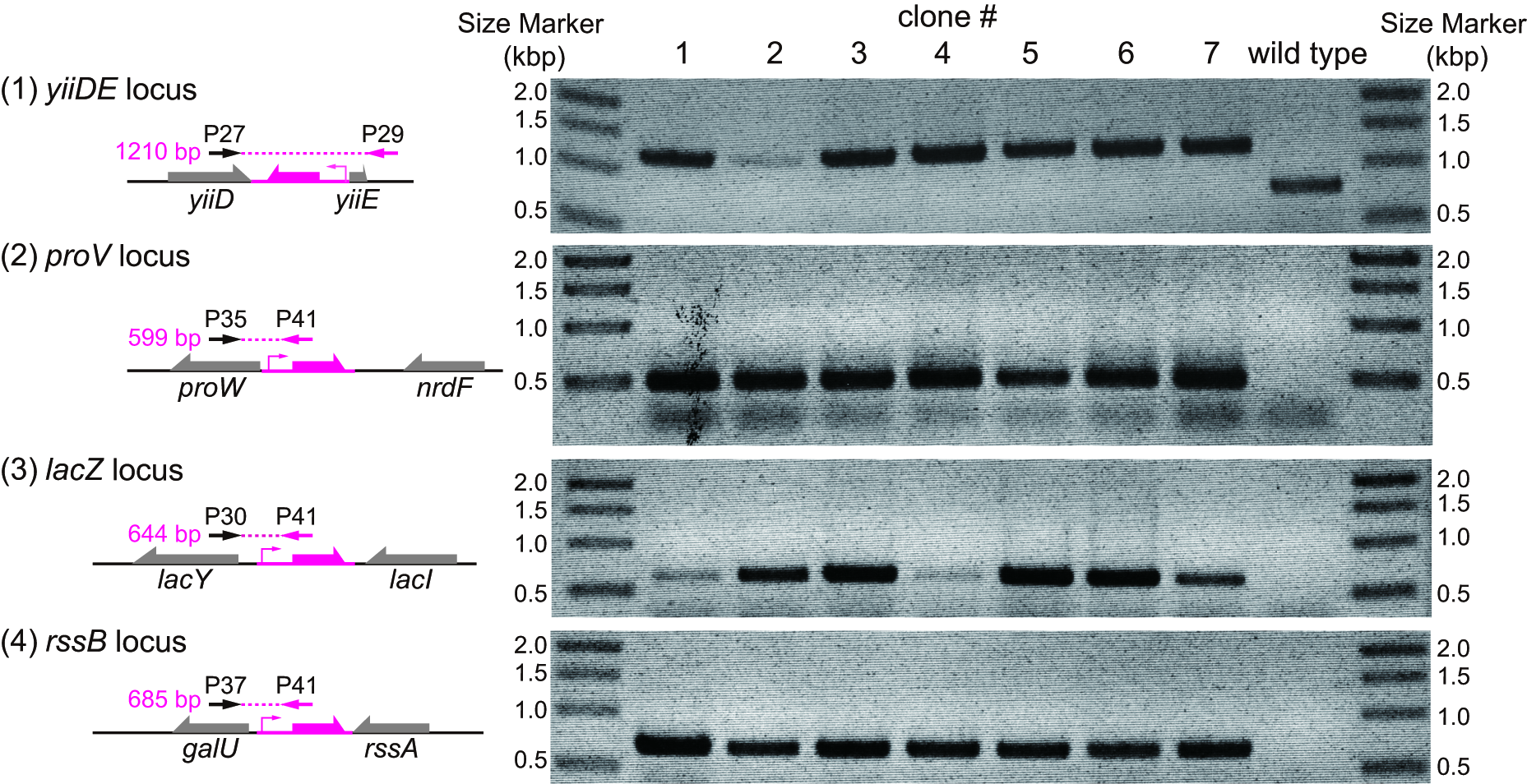

Supplement: S2 Fig — Seven clones were isolated and individually subjected to PCR analysis using primers annealing to the mrfp gene. The local sequence, location of the primers, and expected size (in bp) of the PCR products are shown for the (1) yiiDE, (2) proV, (3) lacZ, and (4) rssB loci. The sequences of the primers used are shown in S1 Table. (TIF) [file pone.0119818.s002.tif]

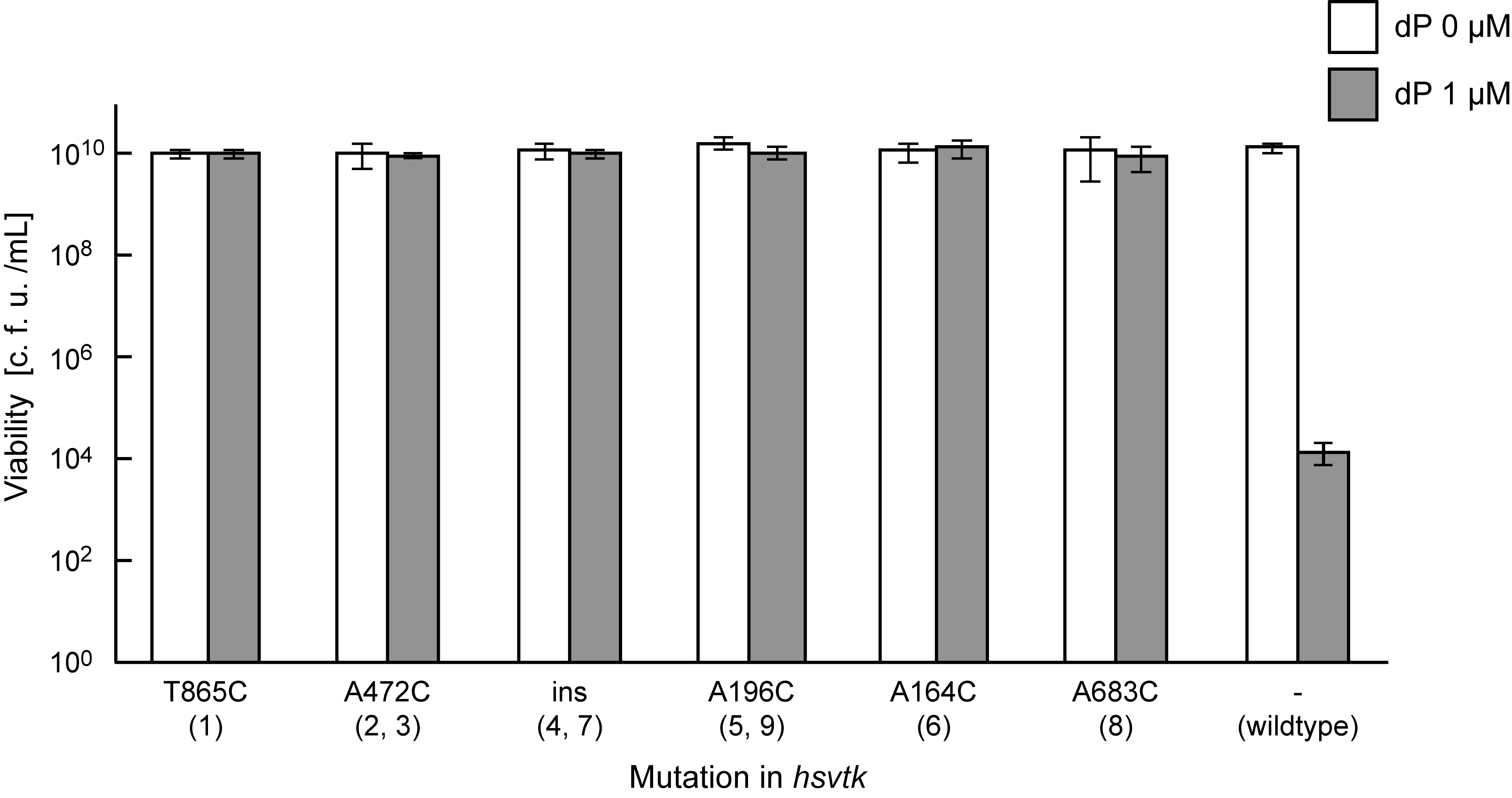

Supplement: S3 Fig — Mutant hsvtk gene identified in dP-selection escapees (S5 Table) were PCR-amplified and inserted into the lacZ locus of MG1655. The resultant recombinants were grown in LB media (0.5 mL) containing Km (50 μg/mL). From each culture, defined number of cells were plated onto LB-Km-agar containing 0 (open bar) or 1 μM (closed bar) of dP. Viability was determined by the colony forming units on each plate. The corresponding clone number in S5 Table is/are also given in parenthesis. (TIF) [file pone.0119818.s003.tif]
