# Supplementary material for: Liquid-Based Iterative Recombineering Method Tolerant to Counter-Selection Escapes
Source: PLoS One. 2015 Mar 16;10(3):e0119818. doi: 10.1371/journal.pone.0119818 (PMC4361647; doi:10.1371/journal.pone.0119818)
Supplement: S1 Table — (PDF) [file pone.0119818.s004.pdf]

Table S1. List of primers used for the experiments in this work.

No	sequence (5'-3')	notes	template DNA
P1	gattcattaatgcagctggcacg	Amplification of HC cassette with *lacZ* homology	pMW-*hsvtk-cat*
P2	ttatttttgacaccagaccaactggtaatggtagcgaccgtgtactgagagtgcacaatatgttacg		
P3	cgatggcggagctgaattac	Amplification of *lacZ* from MG1655 **(Figure 3)**	genomic DNA (MG1655)
P4	gattaatgatcagtggcgcaaagaac		
P5	cctaacagcaccaggatttaaggtgaaattaatctttcatcatacacgagcttcatgcatcg	Amplification of HK/HKH cassette with *yiiDE* locus homology (**Figures 5**)	pHK, pHKH or pJ204-*p_{T5}-mrfp*
P6	ctgatggagaggggcggtgctgcctctctcattcaggtcactggcgatgctgtcggaaac		
P7	atgaccatgattacggattcactggccgtcgttttacaacgagattacgcgcagaccacc	Amplification of HK/HKH cassette with *lacZ* locus homology (**Figures 4, 5**)	
P8	ttatttttgacaccagaccaactggtaatggtagcgaccgcgcccttaggtacgaactc		
P9	cactacccgcagcagggaaataattcccgccaaatagcttgagattacgcgcagaccacc	Amplification of HK, HKH or *pT5-mrfp* cassette with *proV* locus homology (**Figures 4, 5**)	
P10	actgtccgccgctggcgtggtatcccacggattattttgacgcccttaggtacgaactc		
P11	agcatgccactattgagtaaagccagtcaggggagagaacgattacgcgcagaccacctg	Amplification of HK/HKH cassette with *rssB* locus homology (**Figures 5**)	
P12	cattagcaggtaatgcaaatttagcccgcgttatcgtttgcgcccttaggtacgaactc	Amplification of HK/HKH cassette with *rssB* locus homology (**Figures 5**)	

P13	ctttacactttatgcttccggctc	Apmlification of p_L-*gfp*$^{mut3.1}$ cassette with *lacZ* locus homology (**Figure 4**)	pUC-p_L-*gfp*$^{mut3.1}$
P14	gacaccagaccaactggtaatgcatcagagcagattgtactgagag		
P15	cattaccagttggtctggtgtcaaaaataataat		Genomic DNA
P16	gagccggaagcataaagtgtaaag		
P17	cgatggcggagctgaattac		
P18	gattaatgatcagtggcgcaaagaac		
P19	cctaacagcaccaggatttaaggtgaaattaatctttcatgagatctcgatcccgcgaaattaatac	Amplification of p_{T7}-*mrfp* cassette with *yiiDE* locus homology (**Figure 5**)	pET23d-*mrfp*
P20	ctgatggagaggggcggtgctgcctctctcattcaggtcacccattcgccattcagcaaaaaac		
P21	actgtccgccgctggcgtggtatcccacggattattttgagatcccgcgaaattaatacgactcac	Amplification of p_{T7}-*mrfp* cassette with *proV* locus homology (**Figure 5**)	
P22	cactacccgcagcagggaaataattcccgccaaatagcttcccattcgccattcagcaaaaaac		
P23	ttatttttgacaccagaccaactggtaatggtagcgaccggagatctcgatcccgcgaaattaatac	Amplification of p_{T7}-*mrfp* cassette with *lacZ* locus homology (**Figure 5**)	
P24	atgaccatgattacggattcactggccgtcgttttacaaccccattcgccattcagcaaaaaac		
P25	cattagcaggtaatgcaaatttagcccgcgttatcgtttgtcctcaacgacaggagcac	Amplification of p_{T7}-*mrfp* cassette with *rssB* locus homology (**Figure 5**)	
P26	agcatgccactattgagtaaagccagtcagggggagagaacgtcccattcgccattcagc		
P27	gtgtttgaaggcacgtatatcgttc	*yiiDE* test primers	

P28	ctgaacagtgatgtagcaagacacg	*yiiDE* test primers	
P29	atggtttagttatcgcccaggatg		
P30	cagcggttggaataatagcgag	*lacZ* locus sequence/test primer	
P31	gaagaaggcacatggctgaatatcg		
P32	ggatacgacgataccgaagacag		
P33	gattcattaatgcagctggcacg		
P34	gaagagagtcaattcagggtgg		
P35	ctggaagacgggacggaaatg	*proV* test primers	
P36	gcgcgctttagatcgtgagg		
P37	caacggggataacggctttttgac	*rssB* test primers	
P38	ggcaatgccaaatatggggaac		
P39	cggttccaaagcatacgttaaacacc	*mrfp* test primers	
P40	gggaaggacagtttcaggtagtcc		
P41	cgtctttatcctggattacgaccaatc	*hsvtk* test primers	
P42	gtgcaaataaatttaagggtaagttttccgtatg	$gfp^{mut3.1}$ test primers	
P43	gtcggtcttgacaaaaagaaccg	Km^r gene sequence primer	
P44	ctgccgtaccgctcagttgac	$hsvtk_{mod}$ sequence primer	
