# Supplementary material for: Liquid-Based Iterative Recombineering Method Tolerant to Counter-Selection Escapes
Source: PLoS One. 2015 Mar 16;10(3):e0119818. doi: 10.1371/journal.pone.0119818 (PMC4361647; doi:10.1371/journal.pone.0119818)
Supplement: S2 Table — Out of the 92 transformants (chloramphenicol-resistant clones isolated from the pool of MG1655 electroporated with HC cassette), we found three dP-resistant clones. The cassette inserted on chromosome was PCR-amplified using primer P30 and P32 (S1 Table) for the sequence analysis. (PDF) [file pone.0119818.s005.pdf]

Table S2. Sequence analysis of the *hsvtk* gene from dP-resistant clones that arose after the first step of recombination.

Out of the 92 transformants (chloramphenicol-resistant clones isolated from the pool of MG1655 electroporated with HC cassette), we found three dP-resistant clones. The cassette inserted on chromosome was PCR-amplified using primer P30 and P32 (Table S1) for the sequence analysis.

Clone number	Nucleoside substitution	Amino Acid substitution
1	G599A	R200D
2	C123T, G647A	R216H
3	C391T	P131S
