# Supplementary material for: Liquid-Based Iterative Recombineering Method Tolerant to Counter-Selection Escapes
Source: PLoS One. 2015 Mar 16;10(3):e0119818. doi: 10.1371/journal.pone.0119818 (PMC4361647; doi:10.1371/journal.pone.0119818)
Supplement: S3 Table — The HC cassette was amplified with either VentR DNA polymerase or Phusion DNA polymerase using primer P1 and P2 (S1 Table) to attach homology arms for targeting to the lacZ locus. The resultant DNA fragment was electroporated into E.coli strain MG1655 harboring pKD46 and plated onto an LB-Cm and LB-Cm/dP plate. The frequency of dP resistance (%) was calculated by: 100 × [number of colonies observed on Cm/dP plates] / [number of colonies on the Cm plate] The numbers show the average of 3 samples with standard deviations. (PDF) [file pone.0119818.s006.pdf]

Table S3. Frequency of dP-resistant clones in MG1655Δ*lacZ*::*hsvtk-cat* created by HC cassette PCR-amplified using different polymerases.

The HC cassette was amplified with either $Vent_R$® DNA polymerase or Phusion® DNA polymerase ((New England Biolabs) using primer P1 and P2 (Table S1) to attach homology arms for targeting to the *lacZ* locus. The resultant DNA fragment was electroporated into *E.coli* strain MG1655 harboring pKD46 and plated onto an LB-Cm and LB-Cm/dP plate. The frequency of dP resistance (%) was calculated by:

DNA polymerase type	Frequency of dP-resistant clones [%]
Phusion®	0.2 ± 0.1
$Vent_R$®	0.8 ± 0.3

100 × [number of colonies observed on Cm/dP plates] / [number of colonies on the Cm plate]

The numbers show the average of 3 samples with standard deviations.
