# Supplementary material for: Liquid-Based Iterative Recombineering Method Tolerant to Counter-Selection Escapes
Source: PLoS One. 2015 Mar 16;10(3):e0119818. doi: 10.1371/journal.pone.0119818 (PMC4361647; doi:10.1371/journal.pone.0119818)
Supplement: S4 Table — From the first round of two-step recombination using the HK cassette (Table 2), nine clones without GFPmut3.1 fluorescence were individually picked and analyzed in their lacZ (or ΔlacZ::hsvTK-km r) locus. For the sequence of primers used for PCR amplification and sequencing (primers P30, 33, P43, and P44), see S1 Table. (PDF) [file pone.0119818.s007.pdf]

Table S4. Genotype summary of non-fluorescent clones found in the first round of recombineering.

From the first round of two-step recombination using the HK cassette (Table 2), nine clones with no GFP$^{mut3.1}$ fluorescence were individually picked and their *lacZ* (or Δ*lacZ*::*hsvTK-km*^{r}) locus was sequenced. For the sequence of primers used for PCR amplification and sequencing (primers P30, 33, P43, and P44), see Table S1.

clone number	HK cassette	genotype
1	No	mutation in *gfp*$^{mut3.1}$(E205 stop)
2	Yes	mutation in *hsvtk* (D228A)
3	Yes	mutation in *hsvtk* (D228A)
4	Yes	mutation in *hsvtk* (D228A)
5	Yes	mutation in *hsvtk* (T158P)
6	Yes	*hsvtk* missing
7	Yes	*hsvtk* missing
8	Yes	*hsvtk* missing
9	No	No PCR product
