# Supplementary material for: Liquid-Based Iterative Recombineering Method Tolerant to Counter-Selection Escapes
Source: PLoS One. 2015 Mar 16;10(3):e0119818. doi: 10.1371/journal.pone.0119818 (PMC4361647; doi:10.1371/journal.pone.0119818)
Supplement: S5 Table — dP sensitive clone was cultured overnight and plated on dP-selection plates. From these plates nine clones were picked and sequenced in their hsvtk coding regions. (PDF) [file pone.0119818.s008.pdf]

Table S5. Sequencing of the *hsvtk* regions of the counter-selection escapees arose from dP sensitive strain MG1655Δ*lacZ*::*hsvTK-km*^{r}.

dP sensitive clone was cultured overnight and plated on dP-selection plate from which nine clones were picked, and sequenced their *hsvtk* coding regions.

Clone number	Nucleoside substitution	Amino Acid substitution
1	T865C	F289L
2	A472C	T158P
3	A472C	T158P
4	^{865}TTCACCCTG --> ^{865}TTCACCCTG - TTCACCCTG	^{289}FTL --> ^{289}FTL-FTL
5	A196C	T66P
6	A164C	D55A
7	^{865} TTCACCCTG --> ^{865} TTCACCCTG - TTCACCCTG	^{289}FTL --> ^{289}FTL-FTL
8	A683C	D228A
9	A196C	T66P
